# Challenges for the veterinary profession: A grounded theory study of veterinarians' experiences of caring for older horses

**DOI:** 10.1111/evj.14444

**Published:** 2024-11-27

**Authors:** Rebecca Smith, Gina Pinchbeck, Catherine McGowan, Joanne Ireland, Elizabeth Perkins

**Affiliations:** ^1^ Department of Equine Clinical Science, Institute of Infection, Veterinary and Ecological Sciences, Faculty of Health & Life Sciences University of Liverpool Liverpool UK; ^2^ Department of Livestock and One Health, Institute of Infection, Veterinary and Ecological Sciences, Faculty of Health & Life Sciences University of Liverpool Liverpool UK; ^3^ Department of Primary Care and Mental Health, Institute of Population Health, Faculty of Health & Life Sciences University of Liverpool Liverpool UK

**Keywords:** ageing, ethics, health care, horse, horse–human relationships, veterinary medicine

## Abstract

**Background:**

In Great Britain, owners are keeping their horses into increasingly older age, reflecting societal changes in human–animal relationships. The uptake of routine veterinary services is reported to reduce as horses age. Horse owners seek information regarding their animal's health from alternative sources before and/or following veterinary involvement. Information regarding the experiences and behaviours of veterinarians toward older horse health care provision is limited.

**Objective:**

This study sought to explore how veterinary care for the ageing horse is conceptualised and approached by veterinarians treating horses in Great Britain.

**Study design:**

Exploratory qualitative.

**Methods:**

A subset of qualitative data, collected as part of a larger study investigating how owners and veterinarians make decisions regarding the care of the older horses, was analysed using a constructivist grounded theory approach. Data included semi‐structured in‐depth interviews with nine veterinarians and veterinary clinical records pertaining to the horses of 13 participating owners.

**Results:**

Veterinarians valued regular interactions with owners to optimise a horse's management, however, the timing and nature of veterinary involvement varied. The context of older age shaped decision‐making and ‘age‐appropriate’ interventions were negotiated within the context of each horse and owner. Where participants had concerns about a horse, they sought to achieve an owner's adherence to their advice while navigating the veterinarian–owner relationship. Interpersonal dimensions of veterinarian–owner interactions appeared to shape, and could challenge, veterinarians' delivery of health care services and their own sense of being a professional who cares for animals, and about people.

**Main limitations:**

A sample of veterinarians were recruited for this study based on horse owners' involvement.

**Conclusions:**

The provision of veterinary care for the older horse rests upon networks of relationships. Collaboration between the profession and owners in both the design and delivery of, veterinary health care services may better enable different types of knowledge and values to be brought together more effectively.

## 总结

**背景:**

在英国，马主们将马越养越老，这反映了人与动物关系的社会变化。随着马匹年龄的增长，接受常规兽医服务的数量有所减少。兽医介入前/后，马主会从其他渠道寻求有关马匹健康的信息。而关于兽医处理老龄马健康护理方法和经验的信息十分有限。.

**目的:**

本研究旨在探究英国兽医如何理解和应对老年马的兽医护理。.

**研究设计:**

探索性定性研究。.

**方法:**

采用建构主义扎根理论方法，收集定性数据，了解马主和兽医如何就老年马的护理做出决策。数据包括对9名兽医进行的半结构化深入访谈以及13名参与马主的马的兽医临床记录。.

**结果:**

兽医们重视与马主之间的定期互动，优化马匹健康管理，但兽医的参与时机和性质各不相同。马的年龄影响着决策，“适龄”决定了各种措施的制定。根据每匹马和马主不同背景协商制定干预内容。当兽医对马的情况有顾虑时，他们会寻求让马主遵守自己的建议，同时在兽医‐马主关系中主导。兽医与马主之间的人际维度会影响他们提供医疗服务的方式，并可能挑战他们作为关心动物和人的专业人士的身份感。.

**主要局限性:**

本研究中的兽医群体样本是基于马主的参与情况而招募的。.

**结论:**

为老年马匹提供兽医护理依赖于社交关系网络。在设计和提供兽医保健服务时，专业人员与马主之间的合作可能有助于更有效地将不同类型的知识和价值观结合起来。.

## 1 INTRODUCTION

The dependency of domesticated horses on their human counterparts to provide for their needs, as well as the privatised nature of animal health care services, confers a particular network of relationships in which horses and their health are embedded. The uptake of preventive health care measures, such as vaccination and routine veterinary checks, by owners is reported to reduce as horses grow older conferring a reduction in any associated veterinary assessment.^1^, ^2^, ^3^ There are high rates of morbidity and mortality reported in older horse populations.^2^, ^4^, ^5^ This context raises questions about the nature of veterinarians' approaches to the provision of veterinary care and how these may affect the uptake of services by horse owners.

Horse owners are consumers of health care services who can search for, and develop their own ideas about, animal health. Owners are known to use a variety of information sources regarding horse husbandry, and in relation to horse health, veterinarians are reportedly consulted.^6^, ^7^ However, equestrian communities are also generating their own lay repositories of knowledge which are being accessed before and/or following any veterinary input.^8^ This can produce a rapidly changing environment in which a veterinarian's work is being delivered.

The cornerstone of veterinarians' professional role is their requirement to balance the interests of multiple stakeholders. The declaration made on admission to the Royal College of Veterinary Surgeons states that to become, and retain membership of, the profession a veterinarian is required to accept their ‘responsibilities to the public, my clients, the profession and the Royal College of Veterinary Surgeons, and that, ABOVE ALL, my constant endeavour will be to ensure the health and welfare of animals committed to my care’.^9^ Veterinarians' clinical decision‐making appears to be influenced by the owner and animal‐related factors; for example, a focus group study of participants from veterinary practices working in the British racing industry reported that diagnostic procedures (such as endoscopic examination of the horse's airway) were sometimes performed as part of preventive health screening, or without the presence of significant clinical signs due to presentation of the horse by the trainer.^10^ The way in which veterinarians construct veterinary care that is ‘appropriate and adequate’ within the context of veterinary consultation for older horses, and the role of the horse's owner in shaping these approaches, is of importance. Veterinarians' approaches also indicate how they perceive, and balance the interests of stakeholders and can allude to the factors that might be considered to challenge their professional endeavour.

In line with other professions, veterinarians consider their work as specialised, in that it is work which is ‘inaccessible to those lacking the required training and experience’.^11^ Therefore, the aforementioned production of ‘lay’ knowledge has consequences for veterinarians' ideas about the status of their own expertise and has potential consequences for the degree to which their advice is viewed, and adopted, by a horse's owner. While the effect of interactions between patients and providers in shaping health outcomes is widely reported in the human healthcare setting,^12^, ^13^ crucially, there has been little research on veterinarians' experiences or approaches in their professional work of treating older horses. Understanding veterinarians' experiences and how they perceive their professional work to be delivered, or challenged, is therefore of utmost importance for stakeholders interested in horse welfare.

Horse owners are keeping their animals into increasingly old age.^3^ It can be estimated that there are at least 245 000 horses ≥15 years of age across the United Kingdom.^14^, ^15^ This reflects broader societal changes in human–animal relationships with many companion animals being considered family members and cared for into old age.^16^ Therefore, this is a context in which human–horse relationships, old age and ideas about the role of veterinary medicine within the life of the horse can be studied. The aim of this study was to explore how veterinary care for the ageing horse is conceptualised, approached and enacted by veterinarians treating older horses in practices in Great Britain. In doing so, the study sought to develop a theory that explained the influence of veterinarians on veterinarian–owner relationships and older horse health and welfare.

## 2 MATERIALS AND METHODS

### 2.1 Theoretical perspective

The research was underpinned by a social constructionist epistemology.^17^ Constructionist approaches assume multiple realities and seek to understand how these realities are constructed through social processes. This provided a lens for examining the social construction of health and disease in the older horse, and how this is experienced by veterinarians. The study was informed by a symbolic interactionist theoretical perspective. Herbert Blumer was key in the conceptualisation of this perspective and he differentiated it from realist philosophies by the premise that meaning is interpreted by the individual.^18^ Blumer was critical of reductionist approaches to understanding social life, where factors such as money, knowledge, or emotional burden, are used to explain people's actions.^19^ Blumer argues that this so called ‘variable analysis’ is not able to fully capture the complexity of people's experiences.^19^ A symbolic interactionist lens acknowledges the fluidity of knowledge, paying close attention to individual interactions, as well as the wider historical and social context in which those interactions take place.^17^ This approach enabled the study of how people's attitudes, beliefs and decisions regarding the management of an older horse were shaped by past experiences as well as the context in which interactions took place. This study adopted a constructivist grounded theory approach as described by Charmaz to develop a theory that was grounded in people's experiences.^20^ Constructivism is a social scientific perspective addressing how realities are made.^20^ Charmaz's constructivist version of grounded theory allows for the development of multiple core categories and attends to the need to retain participants' own voice, while recognising the interpretive role of the researcher in the construction of novel theory.^20^ Hence, reflexivity of the researchers is a key component in this approach.

### 2.2 Data collection

Qualitative data were collected from veterinarians as part of a larger exploratory study investigating how horse owners and veterinarians make decisions regarding the care of older horses.^21^, ^22^ As part of the wider research project a paired approach to sampling was adopted to gather data from a horse owner and the veterinarian treating that owner's horse. This approach was underpinned by a desire to analyse how veterinarian–owner relationships shaped meaning and action. Horse owners in Great Britain were recruited to take part in the study via online advertisement and word‐of‐mouth. Inclusion criteria required participants to have experience of owning a horse aged ≥15 years. Participants were purposively sampled to initially include those with differing numbers of horses owned, varying housing locations and experience of horse death. Throughout the study theoretical sampling approaches were adopted, such as the adaptation of interview questions to explore developing categories. Veterinary practices were contacted to request clinical records for the particular horse(s) for the previous 2 years. Veterinarians were approached by email or phone call, asked to participate in an interview and provided with a study information sheet. One participating veterinarian was known to Rebecca Smith (RS) beforehand, while all other interviewees had no prior relationship. In total, 25 veterinarians were approached. In some cases, clinical records were obtained but the veterinarian did not take part in the study, or vice versa.

All interviews with veterinarians were undertaken by RS and held between May 2020 and December 2021. The interviews were held remotely, due to restrictions related to the COVID‐19 pandemic, using video‐based software or phone call. Interviews were audio‐recorded using a digital voice recorder and ranged from 45 to 60 min. An interview topic guide (Text S1) was developed based on a review of the literature, analysis of online discussion forums used by horse owners,^8^ and the authors' personal experiences of veterinary work. The questions were phrased in an open manner to enable exploration of participant's own experiences and the topic guide was used in a flexible manner depending on each particular veterinarian and topics that arose during conversations. Field notes were written following interviews and these included notes or impressions about the interactions that had taken place; for example, the ease of recollecting information about a particular owner or horse, or reference to clinical records. Interview recordings were transcribed by a commercial transcription service that uses human transcribers and an intelligent verbatim approach. Transcripts were checked and anonymised by RS.

### 2.3 Data analysis

Data collection and analysis took place concurrently.^20^, ^23^, ^24^ Inductive coding was the first stage of analysis, whereby data were fractured (down to words, phrases or lines), and conceptual labels or ‘codes’ were applied.^20^ When coding, the participant's use of language and the multiple meanings that could be conveyed were considered. Codes that were connected or represented similar concepts in the participant's experience were grouped. Thus, codes were used to develop the categories and the overarching analytic framework. The interview and clinical record data were analysed individually and compared. This contributed to ongoing analysis; for example, the owner's presentation of the issue was found to be pertinent to the veterinarian's experience in both datasets and therefore this was integrated into the developing analysis. The use of constant comparison enabled variation in categories and patterned relationships to be identified. Integrative diagrams were used to examine and document relationships between categories and to generate concepts and tentative theory.^20^ Records of analysis were kept both on paper and, where useful, qualitative data management software (NVivo 12) was used to store the sorted data. The interpretive process continued throughout the course of writing and analysis was ceased at a point where theory was conceptually dense representing the fullness of the participants' experiences. This paper presents a substantive theory, an ‘interpretation or explanation of a delimited problem in a particular area’,^20^ that encompasses three central concepts and a theoretical interpretation of their relationships.

### 2.4 Reflexivity

RS, a female veterinarian with experience of caring for (but not owning) horses, was a PhD scholar trained in social research methods and was primarily responsible for analysis. This experience influenced how RS built rapport with participants during interviews. Being a relative outsider—a veterinarian who had previously worked with small companion animals rather than horses—enabled RS to question participants' use of colloquial language during interviews and question their worldviews during the initial coding process.^20^ To ensure that participants' experiences were represented during the interpretive process RS continually reflected upon her own preconceptions and taken‐for‐granted assumptions about what veterinary practice, and care for an ageing animal, might involve. It was also important to be questioned by and involve the wider research team throughout this process. Regular discussions took place with Elizabeth Perkins, a social scientist with experience in health and social policy research and herself a horse owner, throughout data analysis. Discussions with the wider research team—all veterinarians with expertise in epidemiology and equine medicine—also took place, during which analysis was reviewed and refined.

## 3 RESULTS

In total, nine veterinarians were interviewed (length of time in practice ranged from four to over 20 years, see Table S1 for further participant details) and clinical records pertaining to the horses of 13 participating owners were included. Three central and dynamically interacting concepts and their relationships are presented. First, ‘Engaging with owners and their older horse’ will discuss veterinarians' access to, and their conceptualisations of, the older horse and the factors perceived to shape or to challenge the provision of health care services. Secondly, ‘Negotiating age‐appropriate interventions’ describes how the context of older age shaped decision‐making and the approaches adopted as participants negotiated care within the context of each horse and owner. The final section, ‘Seeking to achieve an owner's adherence to veterinary advice’ outlines how participants framed, and talked about their approaches to, achieving particular management outcomes. The effect of these conceptualisations on approaches toward older horse management and health care is highlighted.

### 3.1 Engaging with owners and their older horse

Participants talked about being reliant on owners seeking their involvement to be able to ‘care for’ a horse. Participating veterinarians worked in practices where they relied upon owners and/or livery staff (in the case of a retirement livery yard) actively seeking veterinary assistance. During the interviews, veterinarians spoke about both their experiences of caring for the participating owner's horse as well as other older horses that they had treated. In general, they were more open to talking about challenges they faced in relation to unspecified owners and their horses.

For the most part, old age for a horse was perceived to represent physical decline and an increased likelihood of disease processes. Approaches to the provision of veterinary care were underpinned by participant's conceptualisations of, and ideas about, the horse's body. The body was perceived to facilitate the performance of daily activities, such as eating, being mobile and interacting with others. While some physical change in a horse's condition might be acceptable in older age, it was the functioning of the body that was important and this factored into how welfare issues were constructed:

I think there's also both the physical and the mental side of it, so obviously physical side of it we've got to look at the body condition score which I think in itself doesn't have to be a problem, can have a thin horse but if that thin horse is stopped from doing other things because it's weak then that might be a, an actual um welfare problem (P1).

Therefore, veterinarians felt it was the owner's responsibility to ‘keep on top of things’, and for an older horse, this meant making accommodations for their changing needs, such as providing appropriate nutrition, pain relief and shelter.

The timing and nature of an owner's presentation of their horse, and their conformity with medicalised veterinary notions of ‘good’ care, shaped the veterinarian–owner relationship. Being a ‘proactive’ owner was believed to involve keeping up with preventive health care measures and employing appropriate professionals in a timely fashion when health problems arose:

I think that Jasper is a patient that I have largely seen just on a routine basis. It has been vaccinations but also his owner was somebody who wanted to get him checked for PPID, or Cushing's disease, because she thought he might have it. So, she was quite a proactive owner (P2).

However, it was difficult for veterinarians when they observed issues in animals not presented for veterinary attention, or when they considered that veterinary involvement was unnecessarily delayed. These apparent delays in advice‐seeking were emotionally upsetting, particularly when horses were presented to them in significant pain.

Participants often alluded to the fact that some owners were unable to detect problems in their horse. This was perceived to be related to negative attitudes toward ageing, a lack of horse‐related knowledge, or limited opportunity to identify issues due to reduced contact with the animal. Owners seen to be delaying advice seeking was perceived to be related to the avoidance of financial input or lack of motivation, and this reflected negatively on the provision of health care:

I had one recently, a horse quidding really badly, stinking, obviously needed serious dental work. If they're quidding, they're in pain. The excuse, “Oh, well he's old. Old horses quid.” Well actually that's not true, that's not true, but I think a lot of excuses are made. I do think finances play a big part in it (P4).

There were some comments made by participants regarding the extent to which owners' approaches to horse health care aligned with what they considered to be ‘appropriate’ care. Issues of horse health and welfare were not necessarily perceived in the same way by owners. Ideas about an owner's ‘level of care’ were also informed by historical knowledge regarding whether their help‐seeking was timely and whether they followed veterinary advice or not. This type of assessment of owners was evident in clinical records, where references such as an owner being ‘good at monitoring’ were documented.

Where veterinary involvement was sought, consultations hinged on identifying problems and finding solutions; the nature of the veterinarian's assessment of the horse's condition was a critical factor in how they approached the consultation. Some veterinarians talked about their role requiring them to raise any issues related to a horse's health or welfare even where consultations were not sought in relation to that issue, or even that particular horse:

well quite importantly I think that partly you should, you should be advising them on where things are getting to be a welfare problem, if the clients don't realise their horse has a welfare problem, then I think you should bring that up (P1).

The nature of each veterinary consultation was produced through the interplay between owner, veterinarian and horse. Veterinary assessment of the horse was influenced by the veterinarian's available time, questions asked or problems raised by owners or livery staff caring for the animal, and practice protocols, for example, whether a dental examination was included as part of a vaccination consultation. The time available to discuss a horse's health was also affected by the workload within the veterinary practice:

depends, honestly, on how busy we are and if we're in a rush, and how much the owner wants to chat. So, in some cases, like I say, the owner might wait and bombard you with 20 questions they've had for two years about the health of the horse. In which case you'd probably have quite a good discussion about the horse's health, care options, management, and you probably get quite a lot of things out of it (P3).

The development of relationships with owners could create opportunities for veterinary involvement outside of formal visits. The means by which owners could seek advice varied between individuals and between practices; for example, in some cases owners could message the practice regarding queries or advice using social media. Providing owners with their mobile number was valued by some participants for the continuity and ability to impact on the animal's care that this form of contact could provide:

So, my mobile is the first point of call. It's a pain, and it is probably one of the aspects that I like least of being a vet. But it is good for continuity. And I think continuity of care in the veterinary side of things is one of the biggest differences to making a difference or not (P5).

However, this could also be challenging due to frequent requests for advice, infringements on personal time, or contact at times when other clients were being seen.

Generally, participants valued regular involvement so that they could input into the horse's management. One veterinarian felt that part of her role was to make sure that routine veterinary visits were perceived to be valuable to the owner, despite the generally ‘problem‐focused’ nature of veterinary consultations:

so, if their horse is only being vaccinated for tetanus, encouraging them to have that visit every year, rather than every two years. And on visits, still making it feel like it's worthwhile, even if actually you haven't picked up a problem (P7).

There were contextual factors that shaped decision‐making and the construction of what might therefore constitute ‘appropriate’ care for a particular horse at a particular time. Participants considered owner‐ and horse‐related factors in their approaches; for example, dental assessment could be scheduled depending on a particular individual context:

When we do go through them, normal practice policy is annual but, given that that is a commercial operation [horse riding establishment], we do select horses for two years, if I think they can get away with it. But a lot of them are on one year, and some are on even shorter, because there are a lot of old mouths (P5).

A number of participants reported that their practice currently, or previously, had offered preventive health care plans. Some of these plans were tailored to older horses and included screening for PPID. However, it was reported that, in general, there was very little uptake by owners. One participant considered that, in the absence of an identified problem, cost was likely to be a factor in this:

I just think because of the cost of a dental plus bloods plus Cushing's bloods plus a vaccination, it came to £200 or something, just people weren't that keen. They said, “Oh, the horse hasn't got a problem, why do I need you,” but it's like, “Well, it might have a problem but you don't know it's got it because we haven't looked at it” (P6).

Therefore, while veterinarians valued regular involvement in an animal's management the way in which services were provided during a consultation varied due to individual and structural factors. This shaped the extent to which discussions about the horse's general health and wellbeing took place. In addition, health care decisions based on the needs of an individual owner and horse could create tensions between ‘ideals’ of preventive healthcare measures (from a professional perspective) and the needs and practicalities for that owner and horse. While this complexity was described by participants, there was primarily a focus on the owner, and their approaches, as being most influential in how the horse was managed. There seemed to be a reliance on the horse's owner to monitor response to treatment and seek further advice if necessary, and this was sometimes documented in clinical records.

### 3.2 Negotiating age‐appropriate interventions

The meaning of ‘appropriate’ care for an older horse was not considered to be clear‐cut because all horses aged differently and individual owners had different approaches. Where a veterinarian was called for a particular concern, or if discussions arose during the consultation relating to a particular concern, the context of old age shaped veterinarians' approaches to making decisions with the horse's owner.

One veterinarian who attended a retirement livery framed her assessment of the horses and negotiation of appropriate care based on the horse's lifestyle:

they might not be 100% sound on whatever dose they're on but as long as they're sort of willing and look like they're moving quite happily we go, well that's alright then (P9).

Assessments of the horse's welfare alongside what was needed for their health also shaped participants' considerations of what might be in the horse's best interests:

with a lot of these older horses if they're coping with it and it's not actually causing them issues well is it fair to put them through the stress of bringing them in and doing things when the teeth naturally fall out within a few years often (P8).

One participant talked about balancing the risks and benefits of surgical interventions in the context of the anticipation of nearing end‐of‐life:

it does change a little bit is when you have to make a big decision such as the horse have say surgery or emergency surgery, you know at some stage we do have to, I might say to a client do you think this is a good idea in a horse of this age, put a horse through this emergency surgery or shall we try and work our way around it in another way um is there some kind of surgery that can be done that doesn't have to be as invasive as the ideal (P1).

The extent to which medical or surgical interventions were deemed to be necessary was related to the assessment of a horse's condition. This was considered alongside an understanding of the owner's goals for, and their access to, a particular lifestyle for their horse as well as perceived attitudes toward further investigations:

He declined [radiographs and nerve blocks] on the basis that the horse would probably end up on long‐term non‐steroidals anyway, which is fair enough. So, we elected to do that. She was put on daily Bute so that she could retain a level of soundness, in, to enable her to do what she is there to do which is just to hack about (P4).

In instances where owners had particular concerns or requested investigation, this could make the navigation of further investigations more straightforward for veterinarians. However, some participants also questioned the usefulness of discussing topics with owners, or performing ‘screening’ tests for issues, that were not understood to be a current problem for the horse:

So, if I see horses and I'm doing a routine and I think it looks a bit odd, I'll suggest that we take bloods. But these practices that take annual bloods every year on a horse… You know, we don't do it on humans, do we? (P5).

Participants acknowledged that owner finances and willingness to pay for different health care interventions had to be considered. One veterinarian reported that owners sometimes needed time to reach a decision about major interventions, and in this process, an owner could change their mind a number of times:

We discussed enucleation, I gave her an estimate and, no, she was going to just call it a day. And then we've ended up taking the eye out, and the pony is happy as Larry. So, that was going to be a hard‐nosed, “No, taking the eye out is too expensive,” and we've still gone and done it. And she seems pleased, because the pony is chuffed to bits (P5).

Participants' views on appropriate care and their understanding of what advice they should provide could also change over time based upon their experiences:

The only occasions I can think of where I kind of feel like I have learnt to make an intervention earlier is older horses that maybe have poorer condition that are getting stuck down, in a field, that lie down and then cannot get up. I have to admit that my attitude towards that is that if they have done it once I am very strong in warning the owners they will do it again. If they do it twice I am even more vocal in my opinion that it is not fair to let them keep going because this will keep happening and you will end up in a situation where you find them, and they have been down all night and then they are really sick. And that is not fair (P2).

This example demonstrates how the presentation of an animal to a veterinarian and their balancing of harms and benefits are related to the extent to which they seek to achieve owner adherence to their advice. Approaches varied depending on the issue, its perceived welfare implications for the animal, and in line with the veterinarian–owner relationship. This is discussed further in the following section.

A multitude of factors shaped the construction of care provision in the context of old age; the owner's concerns and choices, and the veterinarian's approaches. There were reported differences in attitudes regarding the extent of medical interventions that *should* be offered to owners of older horses and views could differ within the same practice. One participant spoke about conflicts that arose between her and her boss when she referred an older horse for colic surgery:

I don't work there anymore, for many reasons. That's one of them. Yes, I got an absolute bollocking. Apparently, I'd wasted everybody's time, wasted the hospital's time, made us all look like fools, gave the owner false hope. I text the surgeon and I was like, “You better fix that.”… He did great. He did absolutely great, the pony. I'm still in touch with his owners now. So he must be 39 now (P4).

In seeking to provide care that they deemed to be appropriate, such challenges faced by veterinarians could, therefore, have wider implications for the veterinary team.

The analysis of clinical records identified variation in documentation of the nature of the consultation, veterinary clinical findings, discussions that took place and management plans. In general, there was no standardised way of making entries, records were problem‐focused and the severity of the problem appeared to influence how much detail was entered. Some records included details of discussions around possible drug side effects or potential worsening of the horse's clinical condition. Concerns over a horse's welfare and discussions with the owner were sometimes documented:

O is aware that long‐term prognosis is guarded (clinical records).

There was evidence of clinical records being used to document decisions where owners might have declined further investigations or treatments, or where one particular course of action had been adopted rather than another:

Also, owner of the view that if not then no more she can do to manage condition and will have to PTS on welfare grounds SO opt to treat and see how responds (clinical records).

One record contained headings to structure entries including: summary of visit, history, examination, treatment and plan. However, records were often incomplete or sparsely populated. Nevertheless, the use of clinical records as an aide memoir during interviews suggests that these documents can prompt further knowledge held by veterinarians regarding particular owners and their horses. Generally, clinical records did not really reflect the complexity described by veterinarians during interviews. They were often written in medical short‐hand and only briefly described communications that had taken place with an owner.

### 3.3 Seeking to achieve an owner's adherence to veterinary advice

Participants generally considered that a horse's owner was the person who knew the horse best however they were not always best placed to see where welfare problems existed. Where veterinarians had concerns about a horse they tried to achieve a balance between encouraging an owner to adhere to their advice while maintaining the veterinarian–owner relationship—and therefore, veterinary input into the horse's management over time. Participants' attempts to achieving adherence to advice by an owner differed depending on the issue and whether it necessitated management change, or whether they felt that euthanasia should be considered.

Some participants talked about trying to get owners to consider the needs of an older horse by encouraging them to appreciate and recognise their horse's experience or health care needs. One means of doing this was to compare the situation to a human experience and the nature of what one might expect to provide for older people:

The thing I say to people, to the owners is: “How many of us, when we reach older years, get out of bed and need a paracetamol every now and …?” You don't run on being perfect every day, yet owners expect their horse to be perfect every day, don't they (P8).

There were examples of participants discussing their own experience in similar cases or drawing upon medical evidence in attempts to encourage management change or end‐of‐life decisions when they considered this to be the best course of action. Conveying understanding of the problem to owners could involve requesting further diagnostic investigations to ‘prove’ their point.

The extent to which the horse's health was categorised as a problem depended upon the impact on a horse's ability to live an age‐appropriate lifestyle. The phrase “quality of life” was said to be used in conversations where there was a perceived welfare concern, often in attempts to prompt an owner to start thinking about end‐of‐life decision making:

Look, she's not looking a lot better, we need to think about quality of life (P6).

However, the use of this term in proactive conversations about the horse's wellbeing was difficult due to the perception that it came with connotations that problems already existed. The role of veterinarians in both supporting care while also providing advice on timely euthanasia decision making was raised as a challenging aspect by one participant. While many participants considered it important for owners to think about quality of life in a positive sense, and thought that it would be good to start conversations early on to promote good welfare, it was not necessarily easy to do this given the negative connotation with the phrase:

I think it would be good to discuss with owners of old horses the things that they should be looking out for, and how to evaluate quality of life, almost before it starts to deteriorate. Or just as it's starting to, so that they've got better markers, and more realistic expectations themselves. But it's easier to do it with clients who are quite engaged, who ask you loads of questions, and you're talking to them quite frequently. But people who you never really see, it's difficult to jump in with a conversation like that (P7).

Depending on the extent of the welfare concern and the nature of the relationship with the owner, the time frames over which participants attempted to achieve adherence could vary. Different strategies were used such as initiating contact with an owner outside of a paid consultation:

I mean, to start off with, phone them back in a week, or 10 days, or something, and just see how they're getting on. See whether things have improved. See whether they're a bit more open to a conversation at that point in time, when it's not quite so much of a shock. Most people haven't thought about, or made plans or decisions, really. Some people have, but most people haven't. Sometimes it all just comes as a bit of a shock when they get told things (P7).

These conversations could span over a number of months or years:

Most owners will come around, I wouldn't say we get—This afternoon, I'm euthanising the oldest horse in the practice. He's got a squamous cell carcinoma on his penis and he had a resection 15 years ago and it has come back and it's horrendous. And I think if we waited another month or two it would have been past the point. But it has been some fairly gentle prodding over a few months, to get them to come to the decision. So, I think it has gone okay, but maybe it could have been done a bit earlier. I mean, that is fairly typical. Most of the time, you can get through to people (P5).

However, one veterinarian who attended a retirement livery felt that owners of those horses were usually fairly open to end‐of‐life decisions if major problems occurred. Perceiving that moving a horse to a retirement livery involved a process in which owners reframed their goals for the horse and this meant they were more prepared to consider end‐of life decisions:

they've kind of already made the decision that if something happens whilst the horse is there that actually that's the end of the day for them (P9).

There was generally an acknowledgement of the emotional attachment between owners and their horse, as well as the significance of the horse in the owner's life. The advice given by veterinarians to horse owners was also said to be affected by their relationship with the owner and how well they knew them. This shaped attitudes and approaches to the management of the horse:

I've got a very aged client at the moment who keeps two aged ponies in a back garden, completely unsuitable. We've got one with chronic laminitis, one with chronic RAO [severe equine asthma], we can't control their symptoms, but trying to tell her to do anything, because these ponies are her lifeline. Her husband died 20 years ago, it's so hard (P6).

you just couldn't storm in and say the poor pony needs to be put to sleep, the pony it's welfare matters but there is the welfare of the owner as well. But I do say to the younger vets sometimes it might seem unreasonable why the owner isn't letting you put something down but just bear in mind it might not be the only reason (P8).

Strategies could be adopted in a step‐wise fashion and veterinarians generally wanted to maintain their relationships with owners to help the horse as best they could.

There was some discussion about the possibility to seek the involvement of other veterinarians, external stakeholders, or authorities if necessary. However, it was suggested that it was uncommon to actually involve these third parties, and unless veterinarians felt this was truly justified on the basis of the horse's welfare, the process was perceived to challenge their professional relationships:

I think you need to, I think there's a bit of client management there, and I think that managing certain clients means these horses suffer for maybe longer than they should, but I just think that's the nature of the beast. Our priority is animal welfare, but you can't make someone do it. Well, you can make someone do it, you get the RSPCA or the police involved, but that's not going to be very politically correct or popular (P6).

Therefore, the meaning of *appropriate and adequate* veterinary care provision for an older horse was individualised. The extent to which owners were afforded autonomy over their animal's management varied and participants could feel a sense of conflicting responsibilities in their relationships with multiple parties. While veterinarian–owner relationships affected the horse's management in varying ways, caring about the owner was also an important part of being a veterinarian. Therefore, participants' knowledge of, and relationship with, the horse's owner influenced their approach when trying to deliver veterinary care to the horse.

## 4 DISCUSSION

The timing and nature of participants' involvement in the care of an older horse was variable. Participants' conceptualisations of ‘good care’ were informed by medicalised views of the ageing animal and these, at times, were perceived to conflict with owners' lay knowledge and beliefs about disease and its management. The horse's owner and their approach to the animal's care informed veterinarians' decision making and was considered key to maintaining horse health over time. Participants drew upon a number of means in attempts to achieve owner adherence to their advice. Interpersonal aspects of veterinarian–owner relationships were perceived to sometimes challenge the delivery of veterinary care that was believed to be optimal for a horse's welfare. This paper discusses potential avenues that the profession may wish to consider with regards to how care in old age might be conceptualised, and service provision adapted, to meet the needs of society and the animals under their care.

Participants' views of old age care were intimately linked to beliefs about how owners should be providing for their horse's needs. This had implications for how veterinarians saw their role in achieving this. While the approaches of veterinarians were shaped by their scientific training and conceptualisations of, and ideas about the horse's body, they were interacting with their clients who were caring for ageing animals within relationships unbounded by the limitations of science. As Sheila Jasanoff suggests, the ways in which we know and represent the world are inseparable from the ways in which we choose to live in it.^25^ There were instances of differing views on the everyday ageing process and what could, or should, be ameliorated through veterinary involvement or intervention. A study of veterinary professionals interviewed about their experiences of treating senior dogs reported beliefs that owners' attribution of changes in their animal to ‘just old age’ may influence owners' awareness and willingness to seek veterinary care, surmising that this was a ‘barrier’ to ‘care’.^26^ However, findings from interviews with owners of older horses identified that an owner's priorities for, and concerns about, accommodating their ageing horse's health care needs were dynamic; changing over time and on an individual basis.^21^, ^22^ This created a spectrum of views around the necessity of certain health care measures, including vaccination.^8^ Furthermore, a focus group study exploring owners' conceptualisations of horse wellbeing found that owners' perceptions were very individualised and this was based on their past knowledge of the horse and the social context in which they lived.^27^ Therefore, there seems to be differing conceptualisations of what it means to care ‘well’ in older age, produced through divergent experiential contexts. In our study, this appears to be affecting veterinarians' everyday experiences as they seek to fulfil their responsibilities to the animals under their care by navigating the veterinarian–owner relationship.

Veterinarians generally recognised that owners knew their horse best but their professional role entailed them being experts in relation to horse health and welfare, and therefore, they valued regular interactions with owners to optimise a horse's management. Participants generally found it easier to discuss issues with owners if they were ‘engaged’ and ‘asked questions’ but found proactive conversations about a horse's quality of life difficult to raise. A study of dog owners reported that owners want to discuss quality of life and suggests that this may be valuable in improving the veterinarian–client relationship.^28^ Literature suggests that interactions between veterinarians and animal owners that resulted in aligned aims for the animal and trust in the veterinarian were found to be important for owners and shaped their decision making.^29^, ^30^ Horse owners' relationships with, and views about the role of, the veterinarian in the care of their older horse were reportedly shaped by past interactions.^22^ Therefore, in this context, veterinarians may benefit from utilising a framework within which to discuss, and plan, the horse's care as they grow older. For humans unable to make autonomous decisions, ‘best interests’ is a key principle through which surrogate decisions for that person are made.^31^ Considerations for decision makers within this framework include; the importance of avoiding discrimination based on age, appearance or condition and the consideration of all relevant circumstances. Multiple forms of knowledge relating to the medical, emotional and relational aspects of a horse's interests may be relevant to health care or management decision making. Therefore, in the context of decisions made for horses by people, the ‘best interests’ concept could be adapted to better enable the knowledge and values of the animal's owner, veterinarian and other parties with an interest in the horse's welfare, to be discussed. Our findings suggest that in addition to such approaches, reflection by veterinarians on the meaning of ‘good’ care in old age may be required to accommodate owners' conceptualisations.

Participants expressed differing views on old age and their attitudes toward ageing animals shaped constructs of health, welfare and treatment decisions. The invasiveness of tests or treatments was a consideration for some participants, and this reflects findings from a study of doctors and medical students treating older patients.^32^ Literature regarding attitudes to the older horse in the veterinary setting is limited. However, a small survey of Austrian veterinarians, reported that factors such as advanced age, perceptions of a fulfilled life, and short life expectancy was said to help veterinarians to deal with euthanasia more easily and calmly.^33^ In our study, euthanasia was commonly discussed in the interviews. Participants raised particular instances where they had difficulties in getting owners to agree to an end‐of‐life decision for an animal in light of their assessment of significant suffering. Within differing human–horse relationships one's views of an animal's experience, of morality and of the ‘right’ course of action, will vary. The veterinary profession constructs euthanasia as ‘painless killing to relieve suffering’.^9^ However, euthanasia decisions may challenge an owner's own sense of responsibility to continue to care for their animal despite ill health. Discourses regarding end‐of‐life planning reflect the individualised nature of such decisions,^34^, ^35^ however the way in which ‘timely’ decision making is constructed as part of an owner's responsibility may still be challenging. The ‘best interests’ concept may be useful in enabling the multiple knowledges, values and contextual factors of people involved in a horse's care to generate relevant considerations for decision making. It may also be beneficial to enhance veterinarians' knowledge of ethical theories and approaches to support decision making if faced with ethical dilemmas in practice.^36^

While veterinarian–owner relationships affected the horse's management in varying ways, caring about the owner was also an important part of being a veterinarian. At times, this could create a sense of conflicting responsibilities if a veterinarian felt that an owner's decision for their animal differed from what might be ‘best’ from a professional perspective. The extent to which animal owners are afforded autonomy over decision making for their animal's health care has raised animal welfare concerns.^37^ There are also discussions around the types of decisions made by veterinarians and whether these are always in the best interests of the animal,^38^ with ethical decision making tools for use in companion animal settings proposed.^39^ Principles‐based approaches provide a useful framework for ethical decision making, however, the derivation of obligations and their use in limiting action remains challenging. While veterinarians have professional obligations to address instances where owners are not meeting their responsibilities of care provision under the Animal Welfare Act,^9^, ^40^ the limits to which owner choice over animal treatment is permissible is not clear cut. It is generally clearer at end of the spectrum where extreme suffering exists. Veterinarians navigate their input into the care of older horses on an individual basis and this can involve veterinarians grappling with complexities, conflicts and emotions within these multiple relationships. We suggest that a focus on understanding how different views regarding animal treatment arise, work on determining interests and how to weigh up conflicting interests within relationship networks, may better serve veterinary decision‐making, veterinarian–owner relationships and horse welfare.

The availability of time framed how veterinarians engaged in discussions with owners. Time limitations were also identified as a prohibiting factor in a study of veterinarians' experiences of discussing horse weight management with clients.^41^ Although increased availability of time for veterinary consultations may enable a shared understanding of values and goals to be explored, this may only be beneficial if the differences in knowledges underpinning these discussions are also acknowledged. Where possible, practice management structures that facilitate continuity in veterinarian–owner interactions will likely also be of benefit. Along with the numerous interactional elements discussed, it may be useful for veterinarians to document the issues and values raised and discussed during a consultation. This may be especially beneficial if different veterinarians are to attend the horse over time. The optimisation of clinical records or computerised systems may be advantageous, both as a site for contextual knowledge to be stored, and as a tool for planning ‘individual patient care and conducting population‐based care’.^42^ These changes must be part of a more holistic approach to improving care outcomes that promote good veterinarian–owner interactions.

This study had some limitations. Veterinarians in the study participated based on their relationship with a particular owner who had volunteered, and therefore, the selection of veterinarians based on some key characteristics was not possible. Recruitment of veterinarians was difficult and the sample size small—however, this is not considered a limitation within the paradigms of qualitative research. There were a few owners who had no veterinarian to nominate, one veterinarian declined to be contacted for interview when the participating owner had mentioned the study to them, and others reported a lack of time as the reason for not participating. The increased demands placed on veterinarians in practice during the COVID‐19 pandemic were also likely to be a major factor. If we are to increase empirical research of veterinarians' experiences, then better understanding of the barriers that exist for participation is required. Nevertheless, participants generally talked about a range of experiences and there was diversity across the sample. The use of remote interviews was convenient for veterinarians who could participate between visits, however rapport building may have been improved in person.^43^ Nevertheless, similar to other researchers using remote methods, rich data were still collected.^44^, ^45^ Further comparative research including veterinarians working in different contexts, such as referral centres or with different horse use such as the sports horse, would enable further variation within concepts to be identified and findings developed.

In conclusion, participants navigated their responsibilities within the context of their daily work and in the frame of reference that they developed through their experience. At times, the worldviews of owners and veterinarians differed. In human health care, approaches to improving health outcomes have sought to better empower individual patients.^42^ However, these approaches have been criticised for their limited recognition of the individualised experience of living with chronic disease, of the social inequalities related to health, and the power dynamics at play in health care interactions.^12^ While a horse's owner remains a key determinant of how a horse is managed in old age, our study suggests that there may be opportunities to improve veterinarians' approaches. A greater sense of collaboration and the use of a framework that serves the multiple types of knowledge and expertise relevant to the horse's welfare in older age may benefit veterinarian–owner relationships and older horses under their care. Nevertheless, this may not be straightforward and power inequalities exist: the veterinarian has power relating to scientific knowledge but the owner has the power of a gatekeeper, to ask other people and to not follow a veterinarian's advice. While a rapid change to any profession is difficult to achieve, in the meantime, owners will continue to create their own ways of and knowledge about how to care for their older horses regardless of the direction the veterinary profession adopts.

## FUNDING INFORMATION

This research was funded by The Horse Trust, grant number G2018.

## CONFLICT OF INTEREST STATEMENT

The authors have declared no conflicting interests.

## AUTHOR CONTRIBUTIONS

**Rebecca Smith:** Conceptualization; methodology; software; data curation; investigation; validation; formal analysis; visualization; project administration; resources; writing – original draft; writing – review and editing. **Gina Pinchbeck:** Conceptualization; funding acquisition; supervision; writing – review and editing. **Catherine McGowan:** Conceptualization; funding acquisition; supervision; writing – review and editing. **Joanne Ireland:** Conceptualization; funding acquisition; supervision; writing – review and editing. **Elizabeth Perkins:** Conceptualization; funding acquisition; supervision; writing – review and editing; formal analysis; methodology; validation.

## DATA INTEGRITY STATEMENT

Rebecca Smith had full access to all the data in the study and takes responsibility for the integrity of the data and the accuracy of data analysis.

## ETHICAL ANIMAL RESEARCH

The research project included voluntary human participants. The study was reviewed and approved by the University of Liverpool Veterinary Research Ethics Committee.

## INFORMED CONSENT

Informed consent was obtained from horse owners to contact their veterinary practice/surgeon to request participation in the study. Informed consent was obtained from participating veterinary surgeons. Written consent was sought. If this was not possible for the participant to complete, verbal consent was gained at the beginning of the interview and stored separately to the interview data.

### PEER REVIEW

The peer review history for this article is available at https://www.webofscience.com/api/gateway/wos/peer-review/10.1111/evj.14444.

## Supporting information


**Text S1.** Interview topic guide.

**Table S1.** Interview participants: Veterinary surgeons.

## Data Availability

The data that support findings of this study are available from the corresponding author upon reasonable request: Open sharing exemption granted by the editor due to lack of provision in the ethics review and consent process.

## References

[1] McGowan TW , Pinchbeck G , Phillips CJC , Perkins N , Hodgson DR , McGowan CM . A survey of aged horses in Queensland, Australia. Part 2: clinical signs and owners' perceptions of health and welfare. Aust Vet J. 2010;88(12):465–471. 10.1111/j.1751-0813.2010.00638.x 21091457

[2] Ireland JL , Clegg PD , McGowan CM , Mckane SA , Pinchbeck GL . A cross‐sectional study of geriatric horses in the United Kingdom. Part 2: health care and disease. Equine Vet J. 2011;43(1):37–44. 10.1111/j.2042-3306.2010.00142.x 21143632

[3] Mueller MK , Sween C , Frank N , Paradis MR . Survey of human‐horse relationships and veterinary care for geriatric horses. J Am Vet Med Assoc. 2018;253(3):337–345. 10.2460/javma.253.3.337 30019999

[4] Ireland JL , Clegg PD , McGowan CM , Platt L , Pinchbeck GL . Factors associated with mortality of geriatric horses in the United Kingdom. Prev Vet Med. 2011;101(3‐4):204–218. 10.1016/j.prevetmed.2011.06.002 21733586

[5] Bushell R , Murray J . A survey of senior equine management: owner practices and confidence. Livestock Sci. 2016;186:69–77. 10.1016/j.livsci.2015.04.024

[6] Hockenhull J , Creighton E . A brief note on the information‐seeking behavior of UK leisure horse owners. J Vet Behav. 2013;8(2):106–110. 10.1016/j.jveb.2012.04.002

[7] Pickering P , Hockenhull J . Optimising the efficacy of equine welfare communications: do equine stakeholders differ in their information‐seeking behaviour and communication preferences? Animals. 2019;10(1):21. 10.3390/ani10010021 31861909 PMC7022754

[8] Smith R , Pinchbeck G , McGowan C , Ireland J , Perkins E . Caring for the older horse: a conceptual model of owner decision making. Animals. 2021;11(5):1309. 10.3390/ani11051309 34063176 PMC8147395

[9] RCVS . Royal College of Veterinary Surgeons code of professional conduct for veterinary surgeons. 2024 Available from: https://www.rcvs.org.uk/setting‐standards/advice‐and‐guidance/code‐of‐professional‐conduct‐for‐veterinary‐surgeons/pdf/

[10] Kinnison T , Cardwell JM . Conflict between direct experience and research‐based evidence is a key challenge to evidence‐based respiratory medicine on British racing yards. Front Vet Sci. 2020;7:266. 10.3389/fvets.2020.00266 32537459 PMC7267464

[11] Freidson E . Part II. The contingencies of professionalism states and associations. In: Freidson E , editor. Professionalism: the third logic. Cambridge, UK: Polity Press; 2001.

[12] Franklin M , Willis K , Lewis S , Smith L . Chronic condition self‐management is a social practice. J Sociol. 2021;59(1):251–231. 10.1177/14407833211038059

[13] Gabe J , Monaghan LF . editor. Key concepts in medical sociology. 3rd ed. Los Angeles, CA: Sage Publications; 2022.

[14] British Equestrian Trade Association . The national equestrian survey 2019. Wetherby: BETA; 2019.

[15] Ireland JL , Clegg PD , McGowan CM , McKane SA , Pinchbeck GL . A cross‐sectional study of geriatric horses in the United Kingdom. Part 1: demographics and management practices. Equine Vet J. 2011;43(1):30–36. 10.1111/j.2042-3306.2010.00145.x 21143631

[16] DeMello M . The pet animal. In: DeMello M , editor. Animals and society: an introduction to human‐animal studies. New York, NY: Columbia University Press; 2012.

[17] Burr V . Social constructionism. 2nd ed. London, UK: Routledge; 2003.

[18] Blumer H . Symbolic interactionism: perspective and method. Englewood Cliffs: Prentice‐Hall; 1969. https://edisciplinas.usp.br/pluginfile.php/2747599/mod_folder/content/0/COMPLEMENTAR%20‐%201969%20‐%20Blumer%20‐%20Symbolic%20Interactionism.pdf

[19] Blumer H . Sociological analysis and the “variable”. Am Sociol Rev. 1956;21(6):683–690. 10.2307/2088418

[20] Charmaz K . Constructing grounded theory. 2nd ed. London, UK: Sage Publications; 2013.

[21] Smith R . Conceptualising older horse care: a sociological exploration of the human‐horse relationship. PhD Thesis. University of Liverpool. 2022.

[22] Smith R , Pinchbeck G , McGowan C , Ireland J , Perkins E . Becoming a matter of veterinary concern. Front Vet Sci. 2024;11:1355996. 10.3389/fvets.2024.1355996 38872799 PMC11169876

[23] Charmaz K . Constructionism and the grounded theory method. In: Holstein JA , Gubrium JF , editors. Handbook of constructionist research. New York, NY: The Guildford Press; 2008. p. 397–412.

[24] Glaser B , Strauss A . The discovery of grounded theory: strategies for qualitative research. London: Aldine Transaction; 2009.

[25] Jasanoff S . In: Jasanoff S , editor. States of knowledge. The co‐production of science and social order. Oxford, UK: Routledge; 2004. 10.4324/9780203413845

[26] Wallis LJ , Radford AD , Belshaw Z , Jackson J , Kubinyi E , German AJ , et al. “Just old age” – a qualitative investigation of owner and veterinary professional experiences of and attitudes to ageing in dogs in the UK. J Small Anim Pract. 2023;64:425–433. 10.1111/jsap.13610 36971187

[27] Smith R , Furtado T , Brigden C , Pinchbeck G , Perkins E . A qualitative exploration of UK leisure horse owners' perceptions of equine wellbeing. Animals. 2022;12(21):2937. 10.3390/ani12212937 36359063 PMC9654126

[28] Hale H , Blackwell E , Roberts C , Roe E , Mullan S . Broadening the veterinary consultation: dog owners want to talk about more than physical health. Animals. 2023;13(3):392. 10.3390/ani13030392 36766281 PMC9913647

[29] Brockman BK , Taylor VA , Brockman CM . The price of unconditional love: consumer decision making for high‐dollar veterinary care. J Bus Res. 2008;61(5):397–405. 10.1016/j.jbusres.2006.09.033

[30] Hughes K , Rhind SM , Mossop L , Cobb K , Morley E , Kerrin M , et al. “Care about my animal, know your stuff and take me seriously”: United Kingdom and Australian clients' views on the capabilities most important in their veterinarians. Vet Rec. 2018;183(17):534. 10.1136/vr.104987 30181131

[31] Mental Capacity Act . Section 4: Best interests explanatory notes. 2005 Available from: https://www.legislation.gov.uk/ukpga/2005/9/notes/division/6/1/2/3?view=plain

[32] Samra R , Griffiths A , Cox T , Conroy S , Gordon A , Gladman JRF . Medical students' and doctors' attitudes towards older patients and their care in hospital settings: a conceptualisation. Age Ageing. 2015;44(5):776–783. 10.1093/ageing/afv082 26185282 PMC4547928

[33] Springer S , Jenner F , Tichy A , Grimm H . Austrian veterinarians' attitudes to euthanasia in equine practice. Animals. 2019;9(2):44. 10.3390/ani9020044 30704140 PMC6406998

[34] Blue Cross . Euthanasia and horses. 2021 Available from: https://www.bluecross.org.uk/horse-end-life

[35] World Horse Welfare . Equine end of life. 2024 Available from: https://www.worldhorsewelfare.org/advice/management/end-of-life

[36] Mullan S , Fawcett A . Veterinary ethics navigating tough cases. Sheffield, UK: 5M Publishing; 2017.

[37] Hiestand KM . The autonomy principle in companion veterinary medicine: a critique. Front Vet Sci. 2022;9:953925. 10.3389/fvets.2022.953925 36246322 PMC9561244

[38] Taylor PM . Just because we can – doesn't mean we should. Equine Vet Educ. 2022;34(4):172–174. 10.1111/eve.13474

[39] Grimm H , Bergadano A , Musk GC , Otto K , Taylor PM , Duncan JC . Drawing the line in clinical treatment of companion animals: recommendations from an ethics working party. Vet Rec. 2018;182(23):664. 10.1136/vr.104559 29602799 PMC6035488

[40] Animal Welfare Act . c.45. 2006 Available from: https://www.legislation.gov.uk/ukpga/2006/45/contents

[41] Lindley G , Pinchbeck G , Furtado T . Equine obesity: Vet client communication. Equine Vet J. 2022;54:26. 10.1111/evj.43_13855

[42] Bodenheimer T , Wagner EH , Grumbach K . Improving primary care for patients with chronic illness. JAMA. 2002;288(14):1775–1779. 10.1001/jama.288.14.1775 12365965

[43] Weller S . (2017) using internet video calls in qualitative (longitudinal) interviews: some implications for rapport. Int J Soc Res Methodol. 2017;20(6):613–625. 10.1080/13645579.2016.1269505

[44] Richens I . Implementation of vaccination strategies on British dairy farms: understanding challenges and perceptions. University of Nottingham; 2015 Available from: https://eprints.nottingham.ac.uk/30439/1/I.%20F.%20Richens%20Thesis%2009.10.2015.pdf

[45] Lynden J , Ogden J , Hollands T . Contracting for care – the construction of the farrier role in supporting horse owners to prevent laminitis. Equine Vet J. 2018;50(5):658–666. 10.1111/evj.12950 29665061

