# Supplementary material for: Challenges for the veterinary profession: A grounded theory study of veterinarians' experiences of caring for older horses
Source: Equine Vet J. 2024 Nov 27;57(4):1053–64. doi: 10.1111/evj.14444 (PMC12135744; doi:10.1111/evj.14444)
Supplement: Supplementary file 1 — Text S1. Interview topic guide. [file EVJ-57-1053-s002.pdf]

Text S1: Interview topic guide

Thank you for taking the time to speak to me today. During this conversation I hope to cover the topics outlined in your information sheet. However, you don't have to talk about anything you don't want to, and if you feel uncomfortable at any point and wish to take a break or end the interview, please let me know. I will be recording the session on a digital voice recorder so that I don't miss anything important, and I may take a few notes during the interview as well. All your responses will be kept confidential and we will ensure that information is anonymised so you cannot be identified.

We know that as a horse gets older they can experience changes in management and in veterinary intervention. In this study we are interested to know more about your approaches and experiences with older horses, to get an idea of what is important to you when caring for these patients.

Horse x and owner x to be substituted with relevant participant names.

Management experiences and vet-owner relationship

- Could you describe your involvement in the care and management of horse x?
- Does horse x have any health or wellbeing issues of concern?
- Do you think that you and owner x have the same views on how to manage these issues?
- How do you think owner x sees your role in the care of horse x? (Prompts: Do you think your advice is valued?)

Horse-human relationship and healthcare

- Do you think that older horses have different care needs compared to younger animals? (Prompts: Could you describe any barriers to older horses receiving appropriate care?)
- How might your priorities and attitudes towards care change when treating an older horse? (Prompts: Would you give certain factors more weight when making decisions?)
- What do you think are the main welfare concerns surrounding older horses? (Prompts: Can you hypothesise as to why these issues may occur?)

Quality of life and euthanasia

- How would you assess quality of life in an older horse? (Prompts: Have your approaches to this changed over time?)
- How do you think owner x assesses quality of life in horse x?
- What do you think the vet's role is surrounding euthanasia decisions in an older horse? (Prompts: How might the vets' expertise be better utilised?)
- What do you think is meant by providing a 'good death' for a horse?

Closing questions

- Is there anything that you might not have thought about before that has occurred to you during this interview?
- Is there anything else you would like to add?
- Is there anything you would like to ask me?

<u>**Veterinary surgeon for horses in a retirement yard**</u>

Questions are in addition to those listed for veterinary surgeons.

Management experiences and vet-owner relationship

- Can you describe your relationship with owner x?
- Can you describe your relationship with yard owner x?
- Are there ways in which this environment alters the decision making process surrounding health or management issues? (Prompts: Could you describe any advantages from your perspective or any barriers to care?)

Quality of life and euthanasia

- Are there ways in which this management system alters the decision making process surrounding euthanasia?
