# Supplementary material for: Challenges for the veterinary profession: A grounded theory study of veterinarians' experiences of caring for older horses
Source: Equine Vet J. 2024 Nov 27;57(4):1053–64. doi: 10.1111/evj.14444 (PMC12135744; doi:10.1111/evj.14444)
Supplement: Supplementary file 2 — Table S1. Interview participants: Veterinary surgeons. [file EVJ-57-1053-s001.pdf]

Equine Veterinary Journal BEVA

Table S1. Interview participants: Veterinary surgeons

Vet number	Veterinary practice description	Role (if not stated then an employed vet)
1	Teaching equine practice, North West England.	
2	Teaching equine practice, North West England.	
3	Private practice - equine department of mixed practice, North England.	
4	Private equine practice, North West England.	
5	Private equine and farm practice, North East England.	Director
6	Private equine practice, Eastern England.	Clinical Director
7	Private mixed practice, South West England.	
8	Private mixed practice - small and large animal departments, East England.	Director
9	Private equine hospital, North West England.	
